# Locus coeruleus-noradrenergic modulation of trigeminal pain: Implications for trigeminal neuralgia and psychiatric comorbidities

**DOI:** 10.1016/j.ynpai.2023.100124

**Published:** 2023-03-20

**Authors:** Basak Donertas-Ayaz, Robert M. Caudle

**Affiliations:** Department of Oral and Maxillofacial Surgery, University of Florida, Gainesville, FL, United States

**Keywords:** Trigeminal neuralgia, Locus coeruleus, Noradrenaline, Neuropathic pain

## Abstract

Trigeminal neuralgia is the most common neuropathic pain involving the craniofacial region. Due to the complex pathophysiology, it is therapeutically difficult to manage. Noradrenaline plays an essential role in the modulation of arousal, attention, cognitive function, stress, and pain. The locus coeruleus, the largest source of noradrenaline in the brain, is involved in the sensory and emotional processing of pain. This review summarizes the knowledge about the involvement of noradrenaline in acute and chronic trigeminal pain conditions and how the activity of the locus coeruleus noradrenergic neurons changes in response to acute and chronic pain conditions and how these changes might be involved in pain-related comorbidities including anxiety, depression, and sleep disturbance.

## Highlights

• The locus coeruleus (LC), the largest source of noradrenaline in the brain, is involved in the sensory and emotional processing of pain.
• Chronic pain results in altered functioning of the LC in rodents.
• Long-term peripheral nerve injury leads to overactivation of LC neurons. These changes are involved in impaired descending pain modulation and pain-related comorbidities such as depression, anxiety, and sleep disorders.
• Evidence regarding the role of the LC in trigeminal neuropathic pain is limited. More studies are needed to explore the role of the LC in trigeminal neuropathic pain.

## 1 Introduction

Trigeminal neuralgia (TN) is a facial pain condition that is defined by the International Headache Society as “a unilateral disorder characterized by brief electric shock-like pains, abrupt in onset and termination, and limited to the distribution of one or more divisions of the trigeminal nerve that typically are triggered by innocuous stimuli” (Headache Classification Committee of the International Headache Society (IHS), 2018). Although clinically well described, the pathophysiology of TN is not fully understood (Sabalys et al., 2013). Based on the existing evidence, the symptoms may arise from neurovascular compression (classical TN) or underlying disease (secondary TN) or may occur without an apparent cause (idiopathic) Headache Classification Committee of the International Headache Society (IHS), 2018)). The estimated annual incidence of TN was reported to be 4 to 13 per 100,000 people (Shankar Kikkeri et al., 2021). TN is more prevalent in women and adults over the age of 40 (De Toledo et al., 2016, Fallata et al., 2017, Siqueira et al., 2009, Tan et al., 2017, Sathasivam et al., 2017, Jainkittivong et al., 2012).

TN imposes substantial health and economic burden on patients, families, and society (Cheng et al., 2017, Tang et al., 2016, Tolle et al., 2006, Allsop et al., 2015). Indeed, TN was reported to reduce quality of life, cause social and occupational impairment, disability (Tolle et al., 2006, Allsop et al., 2015), and psychiatric comorbidities including depression, anxiety, and sleep disorders in patients with TN (Zakrzewska et al., 2017, Devor et al., 2008, Smith, 2013, Wu, 2015, Mačianskytė et al., 2011, Chang et al., 2019). Suboptimal pain management of TN suggests the importance of understanding the detailed mechanisms underlying the pathogenesis to develop novel treatment strategies in TN.

Noradrenaline (NA) plays an essential role in the regulation of cognitive function, sleep/wake state, arousal, attention, mood and stress reactions, and pain (Pertovaara, 2013, Glavin, 1985, Borodovitsyna et al., 2017, Mitchell and Weinshenker, 2010). Most pain research on NA focuses on NA inhibition of pain (Pertovaara, 2013, Llorca-Torralba et al., 2016). However, Taylor and Westlund present a convincing argument that in chronic neuropathic pain NA arising from the locus coeruleus (LC) facilitates pain in supraspinal regions (Taylor and Westlund, 2017). Chronic neuropathic pain results in sustained LC neuronal firing throughout the rostral-caudal distribution of LC fibers (Brightwell and Taylor, 2009). They argue that with continuous NA exposure neurons that process nociception become adapted to the inhibitory functions of NA. The net result is that instead of suppressing nociception the NA becomes part of the pro-nociception feedforward mechanisms that lead to enhanced pain. Thus, understanding NA’s transition from an anti-nociception to a pro-nociception regulatory pathway is likely important in the treatment of TN and associated psychological comorbidities.

To date, most pain studies have concentrated on noradrenergic modulation of spinal nociceptive transmission and the role of NA has been less extensively studied in trigeminal pain. This review summarizes the noradrenergic modulation of acute and chronic trigeminal pain and then addresses the possible involvement of the noradrenergic system in TN-related comorbidities including anxiety, depression, and sleep disturbance.

## 2 Locus coeruleus- noradrenaline system and a brief overview of the trigeminal pain pathway

Seven NA-containing cell groups (A1-A7) provide noradrenergic innervation of the brain and the spinal cord (Dahlstroem and Fuxe, 1964). The A6 cell group, LC, located in dorsolateral pons is the major source of NA in the brain (Benarroch, 2018, Bucci, 2017). Therefore, in this review, the main focus is centered on the LC noradrenergic cell group, and its implications for modulation of trigeminal pain.

**Fig. 1:**
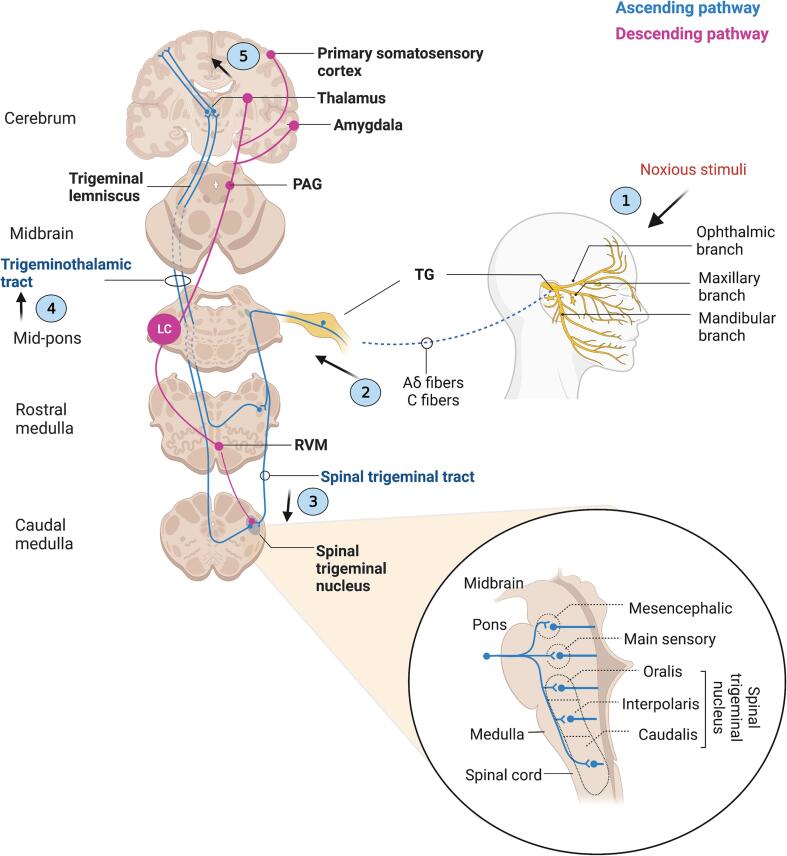
Simplified schematic representation of noxious transmission from face and head to upper brain regions involved in pain modulation. (1) Ophthalmic, maxillary, and mandibular branches of the trigeminal nerve carry noxious sensations from the head and face to the trigeminal ganglion (TG). (2) TG neurons constitute the first-order neurons and nociceptive unmyelinated C and lightly myelinated A-delta fibers coming from the TG are distributed to (3) the trigeminal sensory nuclear complex (TSNC). The TSNC comprises the spinal nucleus and main sensory nucleus. The spinal nucleus consists of three subnuclei: subnucleus oralis, interpolaris, and caudalis. In the TSNC, orofacial nociceptive afferents synapse on second-order neurons, and (4) these neurons then form the ventral trigeminothalamic tract and synapse with third-order neurons in the ventral posteromedial nucleus (VPM) of the thalamus. (5) From the thalamus, nociceptive information is conveyed to the primary and secondary somatosensory cortices. The periaqueductal gray (PAG) and the rostral ventrolateral medulla (RVM) are the two key brain regions that mediate descending pain modulation. The locus coeruleus (LC) receives inputs from the PAG and the RVM and sends inhibitory projections to the TSNC. *Adapted from “Discriminative Pain Pathways”, by**BioRender.com**(2023). Retrieved from**https://app.biorender.com/biorender-templates*.

Ophthalmic, maxillary, and mandibular divisions of the trigeminal nerve (the 5th cranial nerve) carry noxious sensations from the head and face to the trigeminal ganglion (TG) (Maciewicz et al., 1988), see Fig. 1). TG neurons constitute the first-order neurons and nociceptive unmyelinated C and lightly myelinated A-delta fibers coming from the TG are distributed to the trigeminal sensory nuclear complex in the brain stem where they synapse with second-order neurons (Maciewicz et al., 1988). Then, second-order neurons project to the somatosensory and limbic cortices via the thalamus (Maciewicz et al., 1988). The trigeminal sensory nuclear complex consists of the spinal nucleus and main (principal/chief) sensory nucleus (Henssen, 2016). Nociceptive afferents synapse primarily in the spinal nucleus (Maciewicz et al., 1988, Hayashi, 1985, Dessem et al., 2007). The spinal nucleus consists of three subnuclei (subnucleus oralis, subnucleus interpolaris, and subnucleus caudalis) and extends into the upper cervical spinal cord through subnucleus caudalis (also known as medullary dorsal horn) (Maciewicz et al., 1988, Stover et al., 1992, Brown, 1997, Sessle, 2000). The oral nociceptive signal is primarily processed in the principal nucleus, and the subnucleus oralis and interpolaris, while secondarily processed in the subnucleus caudalis, whereas facial nociceptive signals are primarily processed in the subnucleus caudalis (Takemura et al., 2006). Subnucleus oralis is particularly involved in intraoral and perioral nociceptive mechanisms (Dallel et al., 1990). Subnucleus interpolaris contributes to the sensory processing of facial pain (Hayashi, 1985). The subnucleus interpolaris/caudalis transition zone is also involved in deep tissue pain processing (Sugiyo et al., 2005, Wang et al., 2006, Shimizu, 2009, Dubner and Ren, 2004, Ren and Dubner, 2011). In the trigeminal sensory nuclear complex, orofacial nociceptive afferents synapse on second-order wide dynamic range and nociceptive-specific neurons (Maciewicz et al., 1988, Yokota and Matsumoto, 1983, Dubner and Bennett, 1983). These neurons then form the ventral trigeminothalamic tract and synapse with third-order neurons in the ventral posteromedial nucleus of the thalamus (Gauriau and Bernard, 2002, Iwata et al., 1992, Mitchell et al., 2004). From here, the signals are conveyed to the primary and secondary somatosensory cortices (Maciewicz et al., 1988, Sessle and Hu, 1991, Price et al., 2021, Robertson and Kaitz, 1981, Kaitz and Robertson, 1981). Sensory, affective, and cognitive processes modulate nociceptive inputs as they move along the pain pathway at the brainstem and thalamocortical levels (Ossipov et al., 2010).

Sensory information is mainly encoded by the somatosensory cortex, thalamus, anterior cingulate cortex, insula, and periaqueductal gray while emotional responses are mainly encoded by the amygdala, hippocampus, insula, orbitofrontal cortices, and prefrontal cortex (PFC) (for review see (Bushnell et al., 2013). The periaqueductal gray and the rostral ventrolateral medulla are the two key brain regions that mediate descending pain modulation (Chen and Heinricher, 2019). Major sources of afferents to LC are suggested to arise from nucleus paragigantocellularis in the rostral ventrolateral medulla, and nucleus prepositus hypoglossi in the dorsomedial medulla (Aston-Jones et al., 1986). Nucleus paragigantocellularis is linked to cardiovascular, nociceptive and respiratory functions and provides predominantly excitatory inputs to LC (Ennis and Aston-Jones, 1986, Ennis et al., 1992). Nucleus prepositus hypoglossi is involved in the control of eye movements (Aston-Jones, 1991) and it inhibits LC neurons by γ-Aminobutyric acid (GABA) type A receptors in the LC (Ennis and Aston-Jones, 1988, Ennis and Aston-Jones, 1989). Gu et al. also recently showed that LC received projections from the caudal ventrolateral medulla and LC mediated the antinociceptive responses produced by the caudal ventrolateral medulla in mice (Gu et al., 2023).

### 2.1 Effects of noradrenaline under non-pathological conditions

**Fig. 2:**
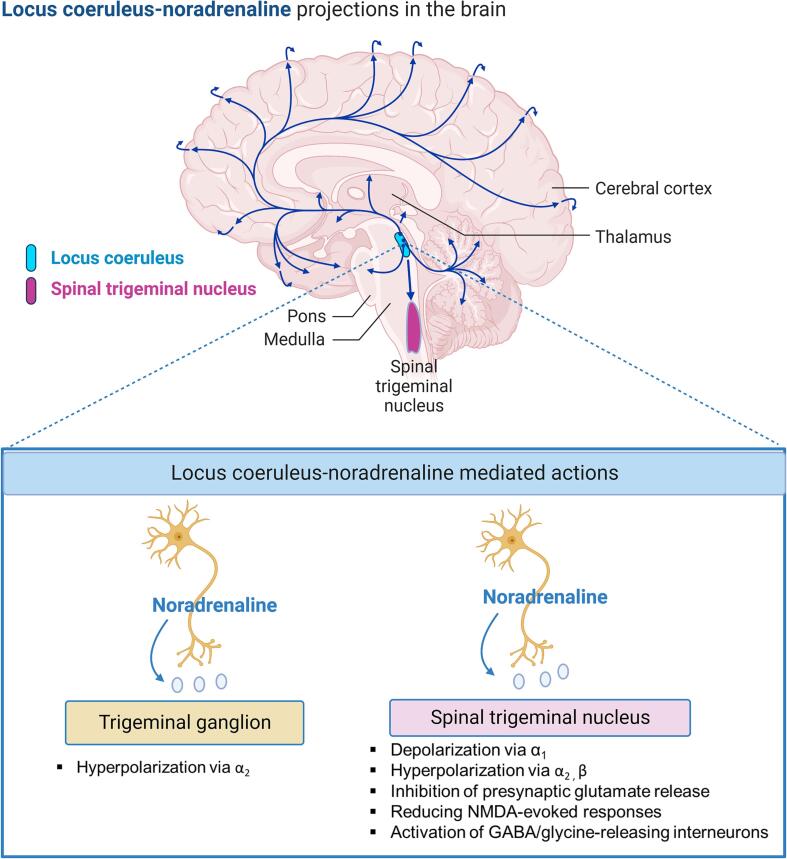
Locus coeruleus-noradrenaline mediated actions in trigeminal ganglia (TG) and subnucleus caudalis. *Adapted from “Distribution of Norepinephrine Neurotransmitters in the Human Brain”, by**BioRender.com**(2023). Retrieved from**https://app.biorender.com/biorender-templates*.

Neuroanatomical experiments show reciprocal pathways connecting LC to trigeminal sensory nuclei (Couto et al., 2006). Activation of the LC neurons results in NA release from the nerve terminals of LC neurons and NA inhibits the sensory transmission in the trigeminal neurons (Sasa and Takaori, 1973, Sasa et al., 1974, Sasa et al., 1977, Matsutani et al., 2000, Sasa, 1979). NA activates G protein-coupled α and β adrenergic receptors (ARs). ARs have three sub-classes each with three receptor subtypes: α1- (α_1A_, α_1B,_ α_1D_), α2- (α_2A_, α_2B_, α_2C_) and β- (β_1_, β_2_, β_3_) (Hieble, 2007, Hieble et al., 1995, Hieble, 1995). Electrophysiological studies investigating the effects of AR agonists in trigeminal sensory pathway showed that several mechanisms mediate the inhibitory actions of NA on primary afferents through activation of ARs. LC-NA mediated actions in TG and subnucleus caudalis are schematized in Fig. 2. NA evokes depolarization of trigeminal subnucleus caudalis neurons via α1-ARs (Han, 2007) while activation of α2-ARs and β-ARs in trigeminal subnucleus caudalis and α2-ARs in TG hyperpolarizes membrane potentials of neurons (Han, 2007, Grudt et al., 1995) by increasing potassium conductance (Grudt et al., 1995) or inhibiting hyperpolarization-activated cation currents (I_h_) (Takeda, 2002) and voltage-gated sodium channel currents (Im, 2018). Activation of ARs also modulates inhibitory or excitatory post-synaptic potentials. Activation of primary afferents evokes excitatory postsynaptic potentials in substantia gelatinosa of the trigeminal subnucleus caudalis mediated by glutamate (Travagli and Williams, 1996) while GABA and glycine interneurons inhibit glutamate-induced depolarization of substantia gelatinosa neurons (Travagli and Williams, 1996). Electrophysiological recordings made from neurons of guinea-pig spinal trigeminal nucleus pars caudalis showed that NA inhibits presynaptic glutamate release by activating presynaptic α2-ARs (Travagli and Williams, 1996) and activates GABA/glycine-releasing interneurons, thereby increasing the frequency of inhibitory postsynaptic potentials (Grudt et al., 1995). In addition, activation of α2-ARs reduced N-methyl-D-aspartate (NMDA)-evoked responses in the medullary dorsal horn of rats (Zhang, 1998). These findings demonstrate that the activation of ARs in trigeminal regions inhibits the sensory transmission in the trigeminal neurons via several mechanisms. However, these mechanisms need to be further investigated in acute and chronic trigeminal pain models.

## 3 Locus coeruleus- noradrenaline system in the modulation of trigeminal pain in rodents

### 3.1 Effects of noradrenaline under acute pain conditions

#### Activation of locus coeruleus produces antinociceptive effects on acute trigeminal nociception

Acute noxious orofacial stimuli activate the descending noradrenergic pathway. As an example, intracisternal administration of capsaicin or experimental incisor tooth movement increased c-Fos immunoreactivity in LC, indicating a change in neuronal activity (Ter Horst et al., 2001, Magdalena et al., 2004, Bullitt, 1990). In another study, infraorbital nerve stimulation enhanced NA levels in the cat’s spinal superfusate, suggesting the activation of a descending noradrenergic pathway (Tyce and Yaksh, 1981). Activation of LC produces antinociceptive effects mediated by NA. For instance, activation of the LC/subcoeruleus neurons via electrical stimulation decreased both noxious (pinch and heat) and non-noxious stimuli evoked responses of rat subnucleus caudalis neurons (Tsuruoka et al., 2003). Iontophoretically applied NA also inhibited noxious heat evoked activity of sensory trigeminal neurons in rats (Cahusac et al., 1995). The evidence from experimental research showed that α2-AR exerts anti-nociceptive effects on acute trigeminal nociception. For instance, microinjection of the α2-AR agonist clonidine into the medullary dorsal horn reduced NMDA-evoked scratching behavior in the facial region (Wang, 2002). Intracisternal and intraperitoneal injection of clonidine produced antinociceptive effects in carrageenan- and formalin-induced orofacial pain (Nag and Mokha, 2016, Yoon et al., 2015), respectively.

#### α2-adrenergic receptor-induced anti-nociception in trigeminal region is modulated by gonadal hormones

Animal studies also showed that α2-AR-induced antinociception is modulated by gonadal hormones. Antinociception produced by activation of the α2-AR in the trigeminal region was attenuated by estrogen in female rats and required testosterone in males (Nag and Mokha, 2016, Nag and Mokha, 2004, Nag and Mokha, 2006, Nag and Mokha, 2009). For instance, intracisternal administration of the α2-AR agonist clonidine into the dorsal part of the medullary dorsal horn produced antinociceptive effects only in intact or testosterone-treated orchidectomized male rats and ovariectomized female rats and α2-AR antagonist yohimbine blocked these effects (Nag and Mokha, 2016, Nag and Mokha, 2009). Sex-specific changes in the α2-AR-mediated inhibition may be one of the factors responsible for the higher prevalence of TN in females and may help us to understand gender and age-associated changes in pain modulation. However, there are conflicting results regarding the role of estrogens in the modulation of facial pain. Contrary to above mentioned studies, aromatase knockout mice which are unable to produce estrogen since birth, had increased nociceptive behavior in the orofacial formalin model and daily estradiol treatment reversed the increase (Multon et al., 2005). Another study with ovariectomized rats showed lower nociceptive threshold to mechanical stimulation applied to the whisker pad area and estrogen replacement increased this threshold (Yu, 2011). These conflicting results may be due to differences in experimental models used, phase of estrous cycle studied. Besides, reported results are based on acute pain conditions, sex-related modulation of α2-AR-induced nociception in the trigeminal region should further be investigated in chronic trigeminal pain studies.

#### Noradrenergic system might be involved in the central sensitization of medullary dorsal horn neurons

Central sensitization refers to increased responsiveness of neurons to non-painful stimuli and is associated with the development and maintenance of chronic pain (Latremoliere and Woolf, 2009). Wang et al., showed that ARs were involved in the central sensitization of medullary dorsal horn neurons. In their study, intrathecal application of an adrenergic antagonist and a sympatholytic compound guanethidine, α-AR antagonist phentolamine, and the α1-AR antagonist prazosin but not the α2-AR antagonist yohimbine attenuated mustard oil-induced trigeminal central sensitization, reflected in increases in mechanoreceptive field size, responses to noxious stimuli, and decreases in activation threshold in nociceptive neurons of subnucleus caudalis (Wang, 2013).

### 3.2 Effects of noradrenaline under trigeminal neuropathic pain conditions

The majority of evidence regarding the effects of LC on neuropathic pain comes from spinal neuropathic pain models. As far as the authors are aware, only one study has directly investigated the involvement of LC in trigeminal neuropathic pain (Kaushal, 2016). In this study, Kaushal et al. demonstrated that the elimination of NA neurons via injection of anti-dopamine β-hydroxylase-saporin into the lateral ventricle and trigeminal brainstem nuclei three weeks after infraorbital nerve injury attenuated mechanical allodynia (Kaushal, 2016). This finding and experimental evidence coming from studies with spinal neuropathic pain (Brightwell and Taylor, 2009, Viisanen and Pertovaara, 2007, Alba-Delgado et al., 2021, Alba-Delgado et al., 2012) suggest that chronic pain may result in altered functioning of pain-modulation circuits including in the LC. Indeed, neuroimaging studies have shown that patients with TN had reduced gray matter volume in various brain regions related to sensory- and cognitive-affective dimensions of pain including the PFC, anterior cingulate cortex, cerebellum, amygdala, periaqueductal gray, insula, thalamus, hypothalamus, putamen, and nucleus accumbens (Zhang, 2018, Tsai et al., 2018, Li et al., 2017, Hayes, 2017). Additionally, patients with trigeminal neuropathy had altered LC functional connectivity with increased connectivity between the rostral ventromedial medulla and decreased connectivity between the ventrolateral periaqueductal gray matter (Mills et al., 2018) which might be related to decreased descending control in the chronic pain patients.

#### Long-term peripheral nerve injury leads to hyperactivation of locus coeruleus neurons

The LC promotes arousal and LC neurons are most active during wakefulness, and their firing rate decreases in sleep (Aston-Jones and Cohen, 2005). During wakefulness, LC-NA neurons fire spontaneously (tonic), and when salient stimuli are presented the tonic firing changes to phasic bursts of activity (Aston-Jones and Cohen, 2005). Rodent studies with spinal neuropathic pain models showed that the firing rates of LC-NA neurons differ at different stages of neuropathic pain. No study investigated the effects of trigeminal neuropathic pain. The spinal studies showed that in the early stages of neuropathy (seven and 14 days after sciatic nerve injury), the tonic activity of LC neurons was preserved (Viisanen and Pertovaara, 2007, Alba-Delgado et al., 2021, Alba-Delgado et al., 2012, Bravo et al., 2014, Alba-Delgado et al., 2012, Llorca-Torralba et al., 2019) while it turned into irregular tonic activity and exacerbated bilateral phasic responses in the long term (28–30 days after nerve injury) (Alba-Delgado et al., 2021, Llorca-Torralba et al., 2019, Alba-Delgado et al., 2013, Alba-Delgado et al., 2016). Phasic activity induces the release of excitatory neurotransmitter glutamate in the LC (Singewald and Philippu, 1998). Peripheral neuropathy increases the excitatory synaptic transmission to activate noradrenergic neurons, and basal extracellular glutamate concentrations in the LC neurons were increased in rats with spinal neuropathic pain (Rohampour et al., 2017, Suto et al., 2014, Kimura et al., 2015). Local glutamatergic and noradrenergic inputs control nerve injury induced glutamate release in the LC (Hayashida et al., 2018). NA evokes hyperpolarization of LC noradrenergic neurons by activating α2-ARs and reduces their firing rate (Aghajanian and VanderMaelen, 1982, Kawahara et al., 1999), while blocking α2-ARs in the LC potentiates the responses of LC neurons to the excitatory stimuli (Simson and Weiss, 1987). Sciatic nerve injury was shown to increase the expression of α2-AR in the LC 28 days after chronic constriction injury (CCI) with no change seven days after nerve injury (Alba-Delgado et al., 2012, Alba-Delgado et al., 2013). It was shown that blockade of α2-AR and group II metabotropic glutamate receptors (mGluRs) in the LC six weeks after spinal nerve ligation induces glutamate release in the LC to activate the descending noradrenergic pathway, reducing hypersensitivity in rats. Concomitant injection of the AMPA receptor antagonist CNQX into the LC dampened these effects (Hayashida et al., 2018). Furthermore, basal GABA levels in the LC increased after spinal nerve ligation in rats (Yoshizumi, 2012) and presynaptic inhibition of GABAergic inhibitory postsynaptic currents in LC neurons of nerve-injured mice produced analgesic effects through activation mediated by the descending noradrenergic system (Takasu et al., 2008). Kaushal et al. also found that the GABA-synthesizing enzyme glutamic acid decarboxylase (GAD65) immunoreactivity increased in the LC after infraorbital nerve injury and the GABA_A_ receptor antagonist bicuculline injected into the LC alleviated mechanical hypersensitivity when the animals were tested at 10 min and 20 min post-infusion (Kaushal, 2016). The LC also receives inhibitory serotoninergic inputs from the dorsal reticular nucleus (Kim et al., 2004). Serotonin was shown to attenuate sensory stimuli evoked responses in the LC (Segal, 1979) and the glutamate-induced excitation of LC neurons (Aston-Jones et al., 1991). Alba-Delgado et al. proposed that inhibitory input from the dorsal reticular nucleus might block the excitatory input from the nucleus paragigantocellularis in the rostral ventrolateral medulla which maintains the constant tonic LC activity in neuropathic pain (Alba-Delgado et al., 2012). Moreover, the LC receives histaminergic innervation from the tuberomammillary nucleus (Panula et al., 1989) which increases the firing rate of LC noradrenergic neurons by activating histamine H1 and H2 receptors (Korotkova et al., 2005). Histamine injection into LC attenuates mechanical hypersensitivity in rats with spinal nerve ligation (Wei et al., 2014). These studies show that noradrenergic activity in LC changes over time after nerve injury and excitatory and inhibitory inputs control the firing activity of the noradrenergic neurons in LC that are involved in descending noradrenergic pain inhibition.

#### Locus coeruleus might be involved in trigeminal neuropathic pain-related comorbidities

Pain affects both sensory and affective responses (Singh et al., 2020). Thus, patients with chronic pain are at high risk of developing emotional disturbances. Anxiety and depression are commonly reported psychiatric disorders in patients with TN (Cheng et al., 2017, Melek et al., 2019). Results from the retrospective cohort studies showed that TN increases the risk of developing depressive, anxiety, or sleep disorder (Wu, 2015). Severe pain and treatment failure are also risk factors of depression and anxiety in those patients (Cheng et al., 2017, Chang et al., 2019).

Animal models of trigeminal neuropathic pain also induce anxiety-like behaviors in rodents. Trigeminal neuropathic pain produced by CCI of the infraorbital nerve was found to cause anxiety-like behaviors in rats and mice evaluated in the open field, elevated plus-maze or light/dark transition tests approximately two weeks after nerve injury, while no depressive-like behavior was observed in the forced swimming test (McIlwrath, 2020, Gambeta et al., 2018, Chen et al., 2021). Trigeminal inflammatory compression injury, another trigeminal neuropathic pain model, has been shown to induce anxiety-like behavior in mice eight weeks after the nerve injury when evaluated in a light/dark transition test. In contrast, no behavioral change was found one or four weeks after nerve injury (Lyons et al., 2015). Trigeminal injury induced by a chronic mental nerve constriction in mice also increased escape/avoidance behavior to the mechanical stimulation (Montes Angeles et al., 2020). Anxiety-like behavior developed at least two weeks after nerve injury and was reported to occur only in CCI rats who developed allodynia (Gambeta et al., 2018), suggesting that as pain develops, nerve injury-induced activation of ascending and descending modulatory pathways can lead to the development of emotional disturbances. Indeed, a neuroimaging study has shown that along with anxiety-like behavior, the neuronal activity of brain regions involved in sensory and emotional aspects of pain were also changed ten weeks after the induction of trigeminal neuropathic pain in rats (McIlwrath, 2020). Dysregulation of monoamine neurotransmitters might be involved in the development of comorbid anxio-depressive behavior. Indeed, decreased levels of NA and its metabolite, vanillylmandelic acid in cerebrospinal fluid were reported in patients with TN (Strittmatter et al., 1997), however, more studies are needed.

#### Altered locus coeruleus function is involved in anxiodepressive symptoms and sleep disturbances in chronic pain

Neuropathic pain induces anxio-depressive behaviors in rodents and changes in the activity of LC noradrenergic neurons might contribute to that. It was shown that sciatic nerve injury provokes anxiety and depressive-like behavior in rats with a concomitant increase in firing rate of the LC neurons and increased expression of α2-AR as well as NA transporter, and tyrosine hydroxylase (Alba-Delgado et al., 2013). Similarly, in another study, neuropathic pain induced by streptozotocin-induced diabetes or CCI of the sciatic nerve was shown to cause anxiety-like behavior in rodents (Alba-Delgado et al., 2016, Sieberg et al., 2018). However, burst firing activity of LC neurons and expression of NA transporter, tyrosine hydroxylase, and phosphorylated cAMP-response element-binding protein (CREB) in the LC showed differences between CCI and streptozotocin-treated rats (Alba-Delgado et al., 2016) where the former increased these measures and the later decreased them. These results suggest that although different types of neuropathic pain provoke anxiety-like behavior in rodents, differential effects of neuropathic pain on noradrenergic activity in LC might result from the differences in the etiology of the neuropathic pain. However, mechanisms and brain pathways underlying neuropathic pain-related psychiatric comorbidities remain unclear and should be explored further.

LC-NA neurons innervate several brain regions involved in anxiety and depression, including the amygdala, hippocampus, PFC (Cha et al., 2016, Hare and Duman, 2020, Jones and Moore, 1977, Li, 2021). Llorca-Torralba et al. demonstrated that long term neuropathic pain induced depressive like behavior in rats with CCI, reflected by increased immobility and decreased climbing and chemogenetic inhibition of the LC neurons projecting to the rostral anterior cingulate cortex reversed this behavior (Llorca-Torralba et al., 20222022). Furthermore, Camarena-Delgado et al. showed that chemogenetic inactivation of the LC projections to dorsal reticular nucleus induced depressive like behavior in naïve rats, however, it did not modify long-term pain-induced depression in rats with CCI of sciatic nerve (Camarena-Delgado et al., 2022).

Llorca-Torralba et al. also demonstrated that the LC-basolateral amygdala (BLA) pathway is involved in the anxiety-like phenotype observed after long-term neuropathic pain, as inhibition of LC neurons projecting to the BLA reversed anxiety in rats with CCI of sciatic nerve while it did not affect the sham treated controls (Llorca-Torralba et al., 2019). Moreover, increasing the firing rate of LC-noradrenergic neurons by photostimulation induced anxiety-like behavior in mice and corticotropin-releasing hormone inputs from the amygdala to the LC mediated this effect (McCall et al., 2015). Optogenetic activation of noradrenergic projections from LC that project to BLA were shown to cause NA release in the BLA and induce anxiety-like behavior mediated by β-ARs (McCall et al., 2017). A neuroimaging study has also demonstrated that patients with TN had decreased gray matter volume in corticolimbic regions, including BLA (Zhang, 2018). Hirschberg et al. also showed that chemogenetic activation of LC-noradrenergic neurons innervating the PFC increased anxiety-like behavior in rats (Hirschberg et al., 2017).

Stress may reflect a part of the mechanism underlying these clinical comorbidities as BLA is a key brain region involved in stress and activity of BLA neurons is modulated by NA-mediated stress responses (Sharp, 2017). For instance, stress-induced activation of LC noradrenergic neurons was shown to increase the firing activity in BLA (Giustino et al., 2020). Moreover, footshock stress was shown to increase the spontaneous firing rate of BLA neurons in rats which was reduced after treatment with the systemic β-blocker propranolol and increased by chemogenetic activation of LC noradrenergic neurons (Giustino et al., 2020). Patients with TN had also increased plasma cortisol and adrenocorticotropin levels (Strittmatter et al., 1996), indicating a stress response. Indeed, persistent pain can be considered as a source of stress and stress is a risk factor for many neuropsychiatric disorders, including anxiety and pain.

In addition to anxio-depressive behavior, the altered activity in LC impairs the sleep-wake cycle. LC-noradrenergic neurons are also crucial for switching between sleep and wakefulness (Takahashi et al., 2010). Neuropathic pain significantly interferes with sleep and patients with TN had a higher risk for developing sleep disorders (Wu, 2015). For instance, nearly 60% of patients with TN reported experiencing occasional awakenings due to pain (Devor et al., 2008) and they were four times more likely to wake up during sleep than people without trigeminal neuropathy (Benoliel et al., 2009). Results from experimental studies also showed that neuropathic pain causes sleep disturbance with an increase in wakefulness and a decrease in non-rapid eye movement sleep in mice with sciatic nerve injury (Ito, 2013, Koh et al., 2015). Neuropathic pain was also shown to increase the activity of LC- PFC noradrenergic neurons in mice with sciatic nerve ligation (Koh et al., 2015) and chemogenetic activation of these neurons exacerbated spontaneous foot-lifts in rats with tibial nerve injury (Hirschberg et al., 2017) and that may be, at least in part, associated with sleep disturbances under neuropathic pain (Koh et al., 2015). These studies suggest that overactivation of LC induced by neuropathic pain might be involved in emotional symptoms and sleep disturbances induced by chronic pain.

### 3.3 Locus coeruleus mediates the analgesic action of drugs tested in animal models of pain

Antidepressants such as the tricyclic antidepressant amitriptyline, and the serotonin-NA re-uptake inhibitor duloxetine are used to relieve neuropathic pain (Obata, 2017). Several animal studies with spinal neuropathic pain showed that noradrenergic descending inhibitory system mediates the action of antidepressants to relieve pain (Hiroki et al., 2017, Ito et al., 2018, Kremer et al., 2018). Antidepressants are thought to restore the impaired noradrenergic descending inhibitory system in chronic pain states (Hayashida and Obata, 2019) and NA and serotonin increase in the spinal cord plays an important role in the analgesic effect of antidepressants in neuropathic pain. For instance, it was found that intraperitoneal injections of amitriptyline or duloxetine attenuate the spinal nerve ligation-induced hyperalgesia and increase the spinal NA/serotonin levels in rats (Hoshino et al., 2015, Matsuoka et al., 2016). As tricyclic antidepressants and serotonin-NA re-uptake inhibitors modulate the neurotransmission both NA and serotonin, the analgesic effects produced in animal models of neuropathic pain do not solely belong to NA. However, it was found that the acute, systemic administration of antidepressants amitriptyline, duloxetine and mirtazapine which affect both NA and serotonin levels have more potent antinociceptive effects than the serotonin-reuptake inhibitor citalopram in rats with CCI of sciatic nerve (Bomholt et al., 2005). Moreover, compounds with greater NA reuptake inhibitory activity are suggested to be more effective for the treatment of pain than compounds having only serotonin reuptake inhibitory activity (Leventhal et al., 2007), supporting the importance of NA in relieving pain.

Animal studies also showed that LC mediates the analgesic effects of various compounds tested in rodent neuropathic pain models. For instance, it was shown that injections of substance P (Muto et al., 2012) or glial cell line-derived neurotrophic factor (Kimura et al., 2015) or morphine (Llorca-Torralba et al., 2018) or histamine (Wei et al., 2014) into LC exert analgesic effects on mechanical allodynia and/or thermal hyperalgesia induced by CCI of the sciatic nerve. Results from the studies with several rodent orofacial pain models also support this notion. It was shown that intraperitoneal injection of carbamazepine, first line-therapy in TN, increases the activity of noradrenergic neurons in the LC of naive rats (Olpe and Jones, 1983). Whisker pad injection of botulinum toxin type A, an alternate therapy in TN, (Morra et al., 2016) reduced the increase in c-Fos expression in LC after formalin-induced orofacial nociception in rats (Matak et al., 2014). Bradykinin injections into the principal sensory trigeminal nucleus and LC produced antinociceptive effect in rats, as assessed by the jaw-opening reflex elicited by the dental pulp electrical stimulation test (Couto et al., 1998) and lesioning of LC with adrenergic neurotoxin N-(2-chloroethyl)-N-ethyl-2-bromobenzylamine (DSP-4) antagonized this effect (Couto et al., 2006). However, it should be noted that as activity of LC-NA neurons changes at acute or chronic pain conditions, future studies are needed to explore the role of LC-NA system in analgesic action of drugs in animal models of acute and chronic orofacial pain conditions.

## 4 Conclusion and perspectives

LC inhibition of nociceptive transmission in acute pain and in long-term neuropathic pain increases the tonic activity of LC-NA neurons. These changes may contribute to impaired descending pain modulation and pain-related comorbidities such as depression, anxiety, and sleep disorders. Although, there is limited evidence on the role of the LC in trigeminal neuropathic pain, the literature supports the involvement of the LC in chronification of pain. However, more studies are needed to explore the role of the LC specifically in trigeminal neuropathic pain.

The LC also, in part, mediates the analgesic effects of antidepressants that inhibit NA reuptake or drugs exerted analgesic effect in several rodent models of neuropathic pain. This suggests that the LC is an important hub in the sensory and emotional integration of pain. Therapies targeting LC to reverse impairment in descending pain modulation on early stages of neuropathic pain might be beneficial to attenuate or prevent the development of persistent pain and related comorbidities.

## Funding

This work was supported by the Facial Pain Research Foundation.

## CRediT authorship contribution statement

**Basak Donertas-Ayaz:** Conceptualization, Investigation, Writing – original draft, Visualization. **Robert M. Caudle:** Writing – review & editing.

## Declaration of Competing Interest

The authors declare that they have no known competing financial interests or personal relationships that could have appeared to influence the work reported in this paper.
